# Improving the quality of care for female rape survivors at Scottish Livingstone Hospital, Molepolole, Botswana: A quality improvement cycle

**DOI:** 10.4102/phcfm.v12i1.2238

**Published:** 2020-06-09

**Authors:** Orleans A. Debley, Zelra Malan

**Affiliations:** 1 Division of Family Medicine and Primary Care, Faculty of Medicine and Health Sciences, Stellenbosch University, Cape Town, South Africa

**Keywords:** quality improvement, women’s health, female, rape, survivor

## Abstract

**Background:**

Rape is prevalent in Botswana, but there has been limited research undertaken to improve the quality of healthcare for female rape survivors in this clinical setting. Research can not only influence the health outcomes of victims but also has the potential to inform policy.

**Aim:**

The aim of this study was to improve the quality of care for female rape survivors in Scottish Livingstone Hospital, Molepolole, Botswana.

**Setting:**

The setting is Scottish Livingstone Hospital, Molepolole, Botswana.

**Methods:**

This study was a qualitative cycle, using the normal steps of performing a baseline audit of clinical practice, planning and implementing changes and re-audit.

**Results:**

A total of 124 patient records were audited, comprising 62 patient records at baseline and re-audit. The mean age of victims was 23 years and the age category with the highest incidence of rape ranged between 12 and 20 years, constituting 47% of patients’ records. During the baseline audit, only one out of 10 structural standards was met, while at re-audit eight structural standards were fully met. Although none of the process standards were met during both audits, statistically significant improvements in performance (*p* < 0.05) were shown in six out of 10 criteria at re-audit.

**Conclusion:**

The quality of care for female rape survivors is suboptimal in our setting. However, simple interventions to improve the structure in place for patients and upskilling the entire practice team to align care to current international standards can improve the overall quality of healthcare.

## Introduction

Globally, one in every 14 women has been sexually assaulted by non-partners.^1^ Thirty-five per cent of women have been assaulted sexually and/or physically by partners or non-partners.^2^ Most of these assaults occur in sub-Saharan Africa, with an estimated 17.4% prevalence in Southern Africa.^1^ In Botswana, it is estimated that one in every 10 women is sexually assaulted during her lifetime.^3,4^ Recently it was shown that rape is a significant crime in Botswana, constituting 4.7% of judicial decisions during 2011.^5^ It is clear that not only is this a serious violation of female rights, but it also highlights the increased demand that the health needs of rape survivors put on existing healthcare facility and services. Violence against women, including rape, is a health priority, necessitating the need for strengthening health systems to address this healthcare problem.^6,7,8,9^ To implement changes, protocols and guidelines must be developed and implemented to capacitate healthcare workers and systems to identify and manage the health needs of female survivors of violence.^7,8,9,10^

Short-term health needs of survivors in the acute phase include prevention of pregnancy, human immunodeficiency virus (HIV) and other sexually transmitted infections (STIs), psychosocial support and the management of physical injuries.^11,12,13^ In the medium to long term, a dedicated follow-up clinic is required for early detection and management of the complications of rape based on individualised patient needs.^11,12,13^ Currently, the prevalence of HIV in Botswana is 17.6%, of which the majority (14.3%) are females.^14,15^ Rape victims are predominantly adolescents who are vulnerable to HIV transmission, which could potentially result in further worsening the current disease burden amongst females. The need for prioritising active HIV preventive interventions is therefore very important.^16^

Long-term health needs include physical and psychological needs and complications. Long-term physical complications include chronic pain, frequent headaches or migraines, gastrointestinal disorders, various gynaecological complaints, hepatitis B infection and cervical cancer. Psychological complications include depression, anxiety, impaired libido, low self-esteem and suicide. Social consequences, for instance, strained relationships or isolation from friends, family, intimate partners and a lower likelihood to marry, are also serious complications.^11,12,13^

Rape survivors may also develop risky lifestyle behaviours, such as engaging in risky sexual behaviour like unprotected sex and promiscuity later on in life.^11,12,13^ Other risky behaviours can include alcohol and substance abuse, unhealthy diet-related behaviours, such as bulimia and anorexia nervosa, as well as delinquency and criminal behaviour.^11,12,13^

In order for the quality of health services provided to improve overall, not only the short- and long-term health needs should be addressed, but also policy guidelines should be in place at the organisational level. According to the World Health Organization (WHO) and Networking HIV/AIDS Community of South Africa (NACOSA) clinical and policy guidelines, quality care includes the health facility having a standard policy or guideline in place. Patients should ideally be attended to within 1 h of arrival.^17,18,19^ HIV and pregnancy tests should be available within 2 h of arrival so that patients are offered post-exposure HIV prophylaxis (PEP), emergency contraception, and/or treatment for STIs when applicable.^17,18,19^

Relevant documentation of the event and thorough examination to gather forensic evidence are mandatory. Referral for psychotherapy and social and legal support should be offered during the initial consultation with the patient. Follow-up is mandatory and must be tailored to the individual patient’s health needs. Follow-up consultation should be used to rule out and manage pregnancy and/or HIV/STIs.^17,18,19^

In terms of structural needs, consulting rooms should be available to offer patients the necessary privacy for initial reassurance and psychological support. Furthermore, there must be a waiting area for family and friends because their presence may be necessary for reassuring the patient.

Upskilling of healthcare providers caring for survivors of rape is a vital component in improving the quality of care. Current guidelines suggest that they undergo standardised evidence-based professional training to enable them to understand and manage the health needs of survivors, as well as to deliver quality care to these patients.^17,18,19^ A literature review conducted by the researcher showed that there is little evidence on the prevalence of sexual assault in Botswana, and that there has not been any published research on improving the quality of care for female rape survivors in the clinical setting. Improving the quality of care for female survivors of rape is therefore of social and scientific importance, not only because of the short- and long-term health-related needs, but also with regard to policy implementation, structural needs as well as upskilling of healthcare providers.

## Aim and objectives

The aim of this study was to improve the quality of care for female rape survivors in Scottish Livingstone Hospital, Molepolole, Botswana. The specific objectives were as follows:

1. to create target standards for care specific for Scottish Livingstone Hospital, Molepolole, Botswana
2. to perform a baseline audit to assess the current quality of care for female rape survivors
3. to plan and implement changes based on standard guidelines to improve the quality of care
4. to re-assess the quality of care after changes have been implemented.

## Methods

### Study design

The quality improvement cycle used the following steps:

1. Create the criteria and measurable target standards.
2. Perform a baseline audit of the quality of care.
3. Perform data analysis and evaluation of performance compared to target standards.
4. Plan changes and implement changes.
5. Re-evaluate the quality of care after the implemented changes.
6. Make further recommendations on improving healthcare for female rape survivors.

### Setting

Scottish Livingstone Hospital is located in Molepolole in the southern part of Botswana. The accident and emergency (A&E) unit of the hospital is where female rape survivors are attended to. Currently, an average of 19 adult female rape survivors attend the clinic monthly. It offers 24-h emergency services and is supported by a 24-h laboratory and pharmacy services. Currently, the clinical psychology and social services are available only on weekdays and on outpatient basis, and there is no dedicated emergency response to rape cases. Female rape survivors are seen at the A&E unit of the hospital together with other emergencies and usually have to wait for their turn in a queue. After patients are registered, they wait to be triaged by a nurse. Baseline laboratory investigations for pregnancy and HIV are collected. They then have to wait to be consulted by the doctor on call, usually when the pregnancy and HIV test results are ready, which takes an average of 4–6 h and sometimes even longer. Doctors and nurses vary in their level of expertise in handling patients. None of the nurses or doctors currently working in the hospital have had any professional training in the care of victims of sexual assault. Some doctors who are not conversant with taking the forensic specimens may defer patient consultation and examination, until another colleague with some experience in this regard is available to offer assistance. There are a total of 82 doctors (15 general practitioners and 13 specialists) and 248 nurses working in the hospital. The A&E unit is staffed with 8 doctors who work in shifts (1 doctor per shift) and 18 nurses (on average 3 nurses per shift). The clinical psychology department consists of one clinical psychologist, four interns and one health assistant, while the social works department consists of four social workers.

Doctors and nurses handling female rape survivors have access to the 2007 Standard National Treatment Guidelines by the Ministry of Health of Botswana.^20^ The structure and process of care for rape patients are captured briefly in this guideline. However, apart from not being detailed and current, there are some important components of the quality of care that has been omitted in the guideline. For instance, the guideline does not recommend the required basic structure needed in place for patient care. It also does not recommend the standard turnaround time for pregnancy and HIV test results or a detailed coordinated follow-up care plan, involving clinical psychology, social and legal services for all patients. Again, it does not highlight standardised professional training for doctors and nurses seeing female rape patients as a key element in quality care for female rape survivors. There is access to the Botswana National HIV and Acquired Immunodeficiency Syndrome (AIDS) Treatment Guidelines as well as a reference manual for health workers on the management of STIs, which meet the standards and recommendations for care in the current guidelines for female rape survivors.^21,22^

This study focused on the services provided by the A&E unit in the acute care setting, as well as the current follow-up care for patients in the outpatient department, including social work and clinical psychology support.

### Study population and sample size

The study population included all female survivors of rape aged 12 years and older. Male rape survivors were excluded from this study because of the disproportionally high prevalence of rape amongst women.^1,2,3,4,5^ Another reason for the exclusion of males from this study was the absence of records for male rape survivors in our local register during the baseline audit except for a few male rape suspects. Exclusion criteria were therefore defined as female rape survivors less than 12 years and all male survivors.

The sample size for this study was calculated with assistance from the Centre for Statistical Consultation (University of Stellenbosch). The sample size was calculated on the basis of a power calculation where the sample size of patients is compared before and after change in clinical practice. A sample size of 62 patients’ records in both baseline audit and re-audit (a total of 124) is required to detect a 20% improvement in the quality of care for female rape survivors, with a power of 80% and a *p*-value of 0.05. The calculation was based on the current level of care, which was considered to be very poor, with less than 10% of female rape survivors receiving standard care demonstrated by a prior review of 10 patients’ records. The review was carried out by the principal investigator on 10 patients’ records attended in the A&E unit between September 2016 and October 2016.

### Involving the practice team

The audit team consisted of three doctors and three nurses who are currently working in the A&E departments (including the medical and nursing heads of the department) and a representative from the hospital quality unit as well as the laboratory, pharmacy, clinical psychology and social works departments. The main researcher was the head of the audit team. Members of the team received training on the evidence-based standard structure, process and outcome criteria for providing quality care for female survivors of rape.^18,19^ The audit team met every month to review the progress of the project, address new challenges and discuss any other issues.

### Setting of criteria and target standards

The audit team decided to use the Networking HIV/AIDS Community of South Africa (NACOSA) guidelines, which is the latest regional evidence-based guideline for providing quality care for female rape survivors in the acute stage of trauma based on internationally accepted evidence.^19^ The guideline addresses the structure and process of care within the Southern African setting in detail and defines the quality of care for female survivors of rape using the following criteria: structural, process and outcome criteria.

#### Structural criteria

Structural criteria were discussed by the audit team, and a structural criteria checklist was developed. A score was assigned to the current level of compliance for each item. The audit team evaluated the structural criteria by inspecting the facility and collectively scored the current level of compliance for each item: 2 for full compliance (structure in place and satisfactorily meets the criteria) and 1 for partial compliance (structure in place but does not satisfactorily meet the criteria and 0 for non-compliance) (see Table 1).

**TABLE 1:** List of structural criteria.

Structural criteria	0	1	2
Availability of trained doctors and nurses competent in providing acute care, and also planning and coordinating follow-up care for rape survivors, based on current evidence-based guidelines	-	-	-
Readily available consulting room for victims	-	-	-
Patients attended to within 1 h of arrival	-	-	-
Availability of comfort packs (underwear, sanitary pads, toiletries, and food – preferably a non-perishable snack pack)	-	-	-
Resource materials with details of possible pregnancy, safe abortion, human immunodeficiency virus (HIV) prophylaxis and sexually transmitted infection (STI) treatment	-	-	-
Availability of emergency contraceptive pills	-	-	-
Availability of post-exposure prophylaxis (PEP)	-	-	-
Availability of STI medications	-	-	-
Special register/records for all cases of rape	-	-	-
Availability of proper filing of all patients records so that they are easily retrieved	-	-	-

#### Process criteria

The target standards for the process were to have 100% of records with documentation of:

1. provision of immediate empathic psychological support within 1 h of arrival as documented in nursing triage notes/doctors plan
2. detailed history and physical examination
3. rapid HIV test and pregnancy test results within 1 h
4. initial dose of post-exposure HIV prophylaxis and emergency contraception administered within 2 h of arrival (unless not indicated)
5. initial dose of STI treatment taken within 2 h of arrival (unless not indicated)
6. collection of forensic samples
7. referral to clinical psychology
8. referral to social services
9. follow-up plan for all patients according to psychosocial and other physical individual patient needs
10. the documentation of each of the 10 items listed above in a patient’s record was scored as 1. The percentage of files with a score of 1 was worked out for each item.

#### Outcomes criteria

The target standards for outcome for patient’s records were to have at least 80% of the following documented (as determined by the audit team because a 100% outcome for each criterion for an initial Quality Improvement cycle (QIC) was considered too high and unrealistic):

1. immediate psychological support
2. detailed history and physical examination
3. human immunodeficiency virus/urine pregnancy test (UPT) within 1 h of arrival
4. post-exposure prophylaxis administered within 2 h of arrival
5. emergency contraception administered within 2 h of arrival
6. appropriate follow-up plan.

### Data collection

For the baseline audit, records of 62 female survivors of rape consulted from December 2016 to May 2017 at the hospital were retrospectively randomly selected from the most recent month. The A&E patients’ register was used and patients’ records were retrieved in batches of 10 by the principal investigator with the assistance of nurses in the audit team. Retrospective data were collected on the basis of the set standards from each patient’s record in order to measure the defined criteria by the audit team. All rape cases attended to during the study period were identified and assigned a study number. These study numbers were then captured electronically and 62 of them randomly selected by selecting every third study number. Data were captured by the principal investigator using Microsoft Excel spreadsheet, with study numbers only after patients’ records were collectively reviewed and audited by the audit team. The structural criteria were evaluated by inspection of the facility by the audit team.

After the implemented changes, another 62 patients’ records were randomly selected, using the same sampling method from January to June 2018 and re-audited using the same audit criteria as in the baseline audit. During the re-audit, a new set of records from rape survivors was audited after the changes had been implemented. These records were then compared with the initial set. The groups were therefore unpaired.

### Data analysis

Data were analysed with the assistance of the Centre for Statistical Consultation at the University of Stellenbosch. Frequencies and percentages were generated at 95% confidence intervals for comparison for significant change between baseline and re-audit. Binary categorical data were summarised in frequency tables and a chi-squared test was used to detect the significant differences (*p* < 0.05).

#### Evaluating the information and planning change

The findings of the initial audit were presented by the principal investigator to the audit team for discussion. The team compared the findings to the current evidence-based standard criteria in order to determine the gaps in our clinical practice. The findings of the audit were also presented by the principal investigator to the entire practice team and management. The gaps in our practice compared to current evidence-based standard criteria were further discussed. Then, the audit team met monthly to review and discuss the realistic implementation of the changes to the current clinical practice agreed upon by the audit team. Plans for change were drawn up over the next 3 months and presented to the entire practice team weekly for a month. The existent standard operating procedure for adult rape survivors was revised to encapsulate our target standards. An algorithm was developed as well and displayed at vantage points within the A&E and outpatient departments as a reminder and quick reference tool for nurses, doctors and other auxiliary staff (Appendix 1).

#### Implementing change

The agreed changes in clinical practice were implemented by the audit team from August to December 2017, with the help of the management and staff involved with care for rape patients in the A&E and outpatient departments. The change consisted of improving the structure by availing space to allow for patient care and education by the practice team so as to align care provided with current international guidelines and recommendations. The standard operative procedure for managing rape was updated and a sexual assault algorithm was developed (see Appendix 1).

#### Re-audit

Data collection, data analysis and interpretation were repeated from January to June 2018 in another group of 62 female survivors of rape after the changes had been implemented. The results from the re-audit were compared with those of the baseline audit to determine whether there were statistically significant improvements (*p* < 0.05) for each criterion, as well as to determine how many of the target standards were met, after the implementation of changes.

Apart from the changes implemented that resulted in some measurable improvements, the re-auditing allowed for further recommendations to be formulated in order to continue improving the quality of care.

### Ethical consideration

Ethical approval to conduct this study was obtained from the Health Research Ethical Committee at Stellenbosch University (reference no. S16/09/170) and the Ministry of Health, Botswana, HPDME 13/18/1 X1 (75). Local permission was also obtained from the Ethics Committee of Scottish Livingstone Hospital (reference no. SLH/PF15).

## Results

### Patient characteristics

A total of 124 patient records were reviewed. Sixty-two patient records were reviewed and analysed in each of the audits, at baseline and re-audit. The median age of the patients was 21 years, and the most affected age category was between 12 and 20 years old, constituting 47% of the total sample (see Figure 1).

**FIGURE 1:**
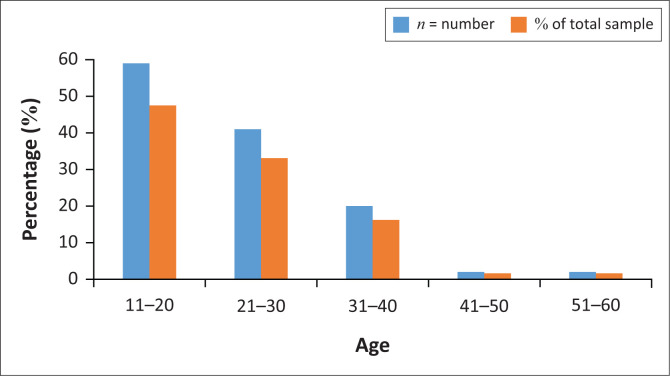
Age distributions of female rape survivors.

### Comparing actual performance with target standards

#### Results of the audit on structural criteria

The results of the audit at baseline and re-audit for structural criteria are compared in Table 2. Only one of 10 structural standards was achieved during the baseline audit. However, during the re-audit, eight structural standards were deemed to have been fully compliant with structural standards. Although the availability of trained doctors and nurses although has not improved compared to the baseline audit, it was still suboptimal considering the current process and outcome performance. Overall, there was a statistically significant improvement in our structural standards, as indicated in Table 2, with a *p*-value of < 0.05.

**TABLE 2:** Results for structural target criteria.

Structure standards	Baseline audit		Re-audit	
	Score	Standard achieved	Score	Standard achieved
Availability of trained doctors/nurses	0	No	1	Yes
Available consulting room for victims	0	No	2	Yes
Triaging of patients within 1 h of arrival	0	No	2	Yes
Availability of comfort packs	0	No	0	No
Availability of resource material on rape	0	No	2	Yes
Availability of start dose of emergency contraceptive pills	0	No	2	Yes
Availability of start dose of PEP	0	No	2	Yes
Availability of sexually transmitted infection prophylaxis	2	Yes	2	Yes
Availability of a rape register	0	No	2	Yes
Availability of files for patients’ records.	0	No	2	Yes

#### Results of the audit on process criteria

The results of the audit at baseline and re-audit for process criteria are compared in Table 3. None of the process standards were achieved at baseline except for completion of forensic kit. However, there was a statistically significant improvement made in all the criteria in the re-audit except for detailed history and physical examination, as well as patients’ referral to social works and clinical psychology for review.

**TABLE 3:** Results of the process criteria.

Process standards	Baseline audit		Re-audit		*p*
	Percentage	Standard achieved	Percentage	Standard achieved	
100% of records with immediate psychological support	8	No	50	No	< 0.001
100% of records with detailed history and physical examination	37	No	48	No	0.138
100% of records with HIV/UPT within 1 h of arrival	13	No	68	No	< 0.001
100% of records with PEP given within 2 h of arrival	0	No	68	No	< 0.001
100% of records with emergency contraception given within 2 h of arrival	0	No	73	No	< 0.001
100% of records with STI prophylaxis given within 2 h of arrival	5	No	87	Yes	< 0.001
100% of records with appropriate referral to clinical psychology	53	No	68	No	0.098
100% of records with appropriate referral to social work	53	No	65	No	0.137
100% of records with forensic kits completed	87	Yes	67	No	0.009

HIV, human immunodeficiency virus; PEP, post-exposure prophylaxis; STI, sexually transmitted infection; UPT, urine pregnancy test.

#### Results on the audit of outcome criteria

Table 4 shows a statistically significant improvement in the quality of care provided to female rape survivors although the target standard of 80% was not achieved. Although our target outcome of 80% was not met in the re-audit as expected, this study showed statistically significant improvement in the provision of immediate non-judgemental empathic psychological support, early administration of post-exposure Highly Active Antiretroviral Therapy (HAART) prophylaxis and emergency contraception as well as appropriate follow-up plan. There was, however, no improvement in documentation of detailed history and physical examination of patients.

**TABLE 4:** Results of outcome criteria.

Outcome standards	Baseline audit		Re-audit		*p*
	Percentage	Standard achieved	Percentage	Standard achieved	
80% records with immediate psychological support	8	No	50	No	< 0.001
80% records with detailed history and physical examination	37	No	48	No	0.138
80% of records with HIV/UPT within 1 h of arrival	13	No	68	No	< 0.001
80% records with PEP administered within 2 h of arrival	0	No	68	No	< 0.001
80% records with emergency contraception administered within 2 h of arrival	19	No	73	No	< 0.001
80% of records with appropriate follow-up plan	19	No	56	No	< 0.001

HIV, human immunodeficiency virus; PEP, post-exposure prophylaxis; UPT, urine pregnancy test.

### Changes to clinical practice

The actual recommendations with changes that were implemented for structural as well as process standards are summarised in Appendices 2 and 3.

## Discussion

### Summary of key findings

The baseline audit confirmed the poor quality of care offered to rape victims as hypothesised. None of the target standards for both structure and process were met during the baseline audit except for the availability of STI prophylaxis. During the re-audit, all the structural target standards improved statistically (*p* < 0.05) except for the availability of comfort packs. Ideally, there should be comfort packs available for patients. However, comfort packs were not available because it is not stocked in the Central Medical Stores (CMS). The hospital financial reserve was strictly for procuring essential drugs and equipment with comfort packs not qualifying for allocation of such funds for now.

None of the target process standards were met in both baseline audit and re-audit; however, a statistically significant improvement was shown in all process criteria except for UPT and HIV test results being ready within 1 h of arrival of patients in the hospital. Improving the turnaround time for getting UPT and HIV test results to 1 h was limited mainly by the lack of test kits. The test kits were usually out of stock during nights and weekends or holiday shifts. During such times, we relied mainly on the hospital laboratory, which is manned by only one staff member during these times. Ironically, those were the times rape cases presented, thereby hampering achieving process standards. However, significant improvements to UPT/HIV test results within 1 h were made.

Although none of the outcome standards were achieved, overall statistically significant improvements were achieved in all outcome criteria. The very poor performance outcomes of the 10 folder pilot study as well as the lack of published local studies contributed to the target aim of 80% being chosen by the audit team for process and outcome criteria. Also, the poor documentation of care given could have contributed to some loss of data, which could have resulted in the unmet outcome. Nonetheless, our study showed that improving the quality of the structure and processes in place for female rape survivors could result in improved quality of care for female rape survivors as supported by a number of other studies.^17,18,19^

### Discussion of the findings in relation to literature and policy

According to the 2013 guidelines published by WHO and the 67th World Health Assembly resolution, there is an urgent need to strengthen the role of health systems in addressing violence against women and girls, including rape. This study demonstrated that improvements were made in the standard of care offered to female rape survivors in the acute stage of presentation as a result of the quality improvement cycle. A new standard operative procedure, training of the practice team as well as an algorithm for managing rape were developed to improve the quality of comprehensive care offered to female rape victims.^6,7,8,9^ Performing rapid HIV/UPTs within the first hour of arrival, early administration of PEP for HIV, providing emergency contraception within 2 h of arrival, as well as appropriate referrals and follow-up plans based on specific patient needs were significantly improved.^17,18,19^

## Limitations

Poor record-keeping and the lack of proper filing delayed the baseline audit as patients’ records had to be retrieved from the general pool of patient’ records. Test kits for urine pregnancy and HIV were usually out of stock during most periods of our study. Although it was discussed during our monthly audit meetings, the quality improvement procedures by the hospital quality department and accreditation team recommended that we used only stocks strictly procured by the pharmacy preferably from the CMS of the Ministry of Health, Botswana.

There was also poor documentation and often poor legibility of what was documented. As this study was mainly a documentation audit, it meant that activities not recorded and that were not legible did not meet the audit criteria, thereby possibly resulting in missing data. Besides, there was generally poor adherence of staff to some of the recommendations proposed by the audit team amongst both nurses and doctors despite the repeated reminders often given.

## Recommendations and implications

Significant improvements in our current performance can only be achieved and maintained through an ongoing quality improvement cycle. The entire hospital staff and management must be motivated by and committed to the provision of an improved quality of care to patients.

The structural procedure needs further training of all nurses and doctors. The audit team will be meeting the Superintendent of Botswana Police Services with regard to informing patients on the need of comfort packs and allowing them to arrange for their own comfort packs for now.

The process criteria require access to pregnancy and HIV test kits always. When out of stock, provisions must be in place so that it could be procured from an accredited supplier as often they are very affordable.

The audit team recommends that this audit should be extended to other facilities within the district and, if possible, the entire nation so that more rape victims could benefit from an improved quality care. This study can also become the basis upon which the current Botswana Standard Treatment Guidelines for Rape can be updated.

## Conclusion

The quality of care provided for female rape survivors was suboptimal in our setting as indicated by the baseline audit. Simple interventions were designed and implemented, which resulted in statistically significant improvements in the quality of care provided to female rape survivors despite the fact that none of the outcome targets were achieved in the re-audit. It is therefore recommended that the quality of improvement process is continued to ensure that improved outcome targets are achieved and the improvements also achieved are maintained.

## Figures and Tables

**FIGURE 1-A1:**
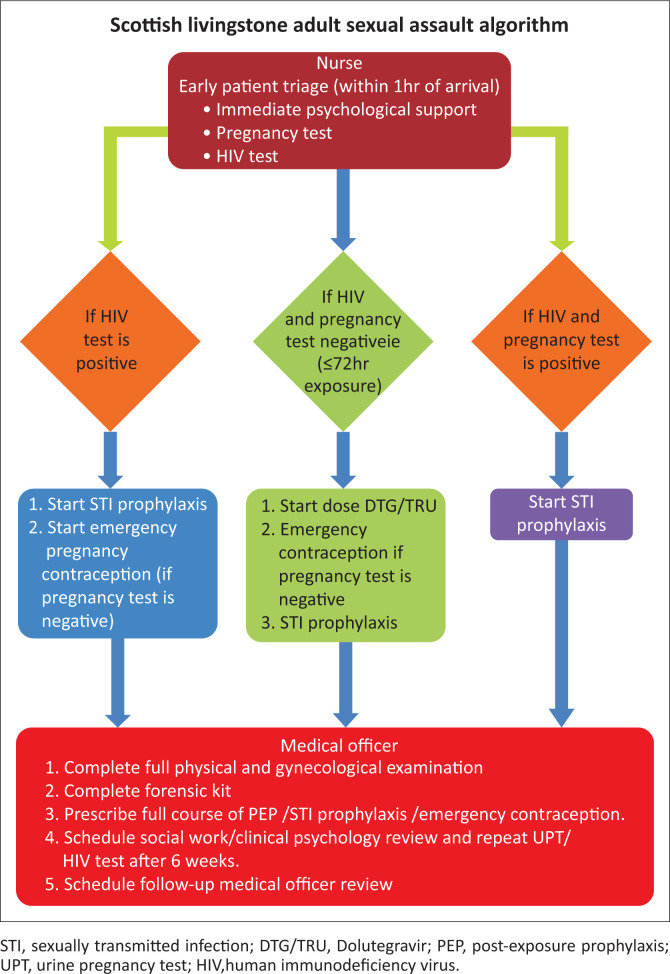
Sexual assault algorithm.

## Appendix 2

**TABLE 1-A2:** Recommendations and actual changes for structural standards.

Structure standards	Recommendation of audit team	Action taken
Availability of trained doctors and nurses	The knowledge and practices of all nurses and doctors attending female rape survivors must be updated to current evidence-based guidelines.	Continuous in-service training facilitated by main researcher as well as nurse in-charge on the management of female rape survivors based on current evidence-based guidelines was held for all nurses and doctors in the hospital attending to victims of rape. A sexual assault algorithm was developed and displayed at vantage points in the hospital where rape victims are attended to.
Available consulting room for victims	Nurse in-charge to ensure that a room which was safe and private was dedicated to rape victims at all times.	The cleaners and orderlies in the department were assigned to the cleaning and re-arranging one of the rooms agreed upon by the audit committee which is used as a counselling room.
Triaging of patients within 1 h of arrival	Nurses to ensure that the rape victims are given as much priority as possible and triaged immediately.	Audit meetings were held monthly on the first Thursday and nurse in-charge assigned to remind all nurses to highly prioritise the care of rape victims. Triage forms were also reviewed weekly to ensure that nurses were adhering to guidelines in this regard.
Availability of comfort packs	To be ordered by the nurse in charge.	Audit meetings were held monthly on the first Thursday of the month, and the nurse in-charge assigned to follow-up on procurement of comfort packs.
Availability of resource material on rape	Principal investigator to ensure that there was a comprehensive leaflet (titled ‘Rising from the Ground’) on the complications of rape and the clinical interventions available in the hospital.	The audit ensured the availability of the ‘Rising from the Ground’ leaflets at all times in the accident and emergency department.
Availability of start doses of post-exposure prophylaxis/ emergency contraception and STI prophylaxis for administration within 2 h of patients’ arrival	Principal investigator to develop a new standard operating procedure (SOP) on sexual assault that will be endorsed by the audit team, which will specify the administration of post-exposure HIV prophylaxis/ emergency contraception and STI prophylaxis within 2 h of arrival of rape victims to the hospital.	The new SOP on sexual assault was forwarded to the pharmacy department. A special order and stocking of post-exposure HIV prophylaxis, emergency contraception and STI prophylaxis was made available for purposes of use only for rape victims in the A&E unit.
Availability of a rape register	Nurse in-charge should order a new register for all cases of sexual assault.	The nurse in-charge obtained a separate register for all female rape survivors. This was reviewed and verified by the audit team during meetings which were held monthly on the first Thursday of the month.
Availability of proper filing of rape records	Also duplicate copies of triage forms should be properly filed by the nurse in-charge.	The nurse in-charge assigned an auxiliary staff to collate all medical records of rape victims (mainly duplicates of triage forms) and appropriately file them. This was reviewed and verified by the audit team during meetings which were held monthly on the first Thursday of the month.

## Appendix 3

**TABLE 1-A3:** Recommendations and actual changes for process standards.

Process standards	Recommendations of audit team	Action taken
100% of patients’ records with HIV/UPT results within 1 h of arrival	Nurse in-charge should order sufficient urine pregnancy test (UPT) kits. A copy of the monthly duty roster with contact details of ITECH personnel to be distributed to A&E unit for urgent Refuse Hospital Treatment (RHT) testing.	Each triage nurse should do UPT immediately after checking patients’ vital signs and thereafter ensure that HIV test of the victim is resulted within 1 h by ITECH personnel. Both HIV/UPT is now a standard part of triaging for rape victims. This was constantly reviewed and re-emphasised by the audit team during meetings held monthly on the first Thursday of the month.
100% of patients’ records with immediate psychological support	Each nurse on call should be able to empathically reassure and promptly triage victims of rape.	In-service training with simulations and peer reviews were held for all nursing staff in the A&E unit. Retraining was done quarterly. This was also constantly reviewed and re-emphasised by the audit team during meetings which were held monthly on the first Thursday of the month.
100% of patients’ records with PEP given within 2 h of arrival	Nurse in-charge to ensure sufficient stocking of start doses of PEP (truvada/dolutegravir) at all times in the A&E unit.	Start dose of PEP should be administered by triage nurse to rape victims with negative HIV test result unless otherwise contraindicated within 2 h of arrival. This was reviewed and re-emphasised by the audit team during meetings monthly which were held on the first Thursday of the month.
100% of patients’ records with emergency contraception given within 2 h of arrival	Nurse in-charge to ensure sufficient stocking of emergency contraception (nordette/norethisterone) at all times in the A&E unit.	Start dose of emergency contraception should be administered by triage nurse to rape victims with negative urine pregnancy test result unless otherwise contraindicated within 2 h of arrival. Again, this was reviewed and shortfalls were addressed by the audit team during meetings which were held monthly on the first Thursday of the month.
100% of patients’ records with STI prophylaxis given within 2 h of arrival	Nurse in-charge to ensure sufficient stocking of STI prophylaxis (ceftriaxone/azithromycin and metronidazole) at all times in the A&E unit.	Start dose of STI prophylaxis should be administered by triage nurse to all rape victims within 2 h of arrival. This was reviewed and shortfalls were discussed and addressed by the audit team during meetings which were held monthly on the first Thursday of the month.
100% of patients’ records with appropriate referral to a clinical psychologist	Doctor on call must routinely refer all rape victims for review by a clinical psychologist.	Discharging nurse should verify that doctor-on-call’s discharge plan includes referral to a clinical psychologist and legal services when indicated before discharging any rape victim. This was reviewed and shortfalls were addressed by the audit team during meetings which were held monthly on the first Thursday of the month.
100% of patients’ records with appropriate referral to a social work	Doctor on call must routinely refer all rape victims for review by a social worker.	Discharging nurse should verify that doctor-on-call’s discharge plan includes referral to a social worker before discharging any rape victim. This was reviewed and shortfalls were addressed by the audit team during meetings which were held monthly on the first Thursday of the month.
100% of patients’ records with appropriate follow-up plan	Doctor on call must routinely have a thorough follow-up plan tailored to individual patients’ need including managing pregnancy and abortion services.	Discharging nurse should verify that doctor-on-call’s discharge plan includes a follow-up review date with a doctor. Shortfalls were reviewed and addressed by the audit team during meetings which were held monthly on the first Thursday of the month.
100% of patients’ records with appropriate referral to legal services	Doctor-on-call must routinely refer all rape victims for legal services, which includes taking forensic specimen and appropriately completing all relevant forms.	Discharging nurse should verify that doctor-on-call’s discharge plan includes documentations of completing forensic kit and appropriately completing all medico-legal forms. This was reviewed and shortfalls were addressed by the audit team during meetings which were held monthly on the first Thursday of the month.
